# The Triple T Allergy Hypothesis

**DOI:** 10.1080/10446670410001722258

**Published:** 2004-06

**Authors:** Matthias Wjst

**Affiliations:** Gruppe Molekulare Epidemiologie Institut für Epidemiologie GSF - Forschungszentrum für Umwelt und Gesundheit Munich Germany

## Abstract

The early induction of allergy is a complex process involving protective and destructive gene variants, environmental and nutritional co-factors as well as allergen exposure. Although critical doses, interactions and susceptible time frames have not been identified so far, late gestation and early childhood seem to be important time periods for allergic sensitization.

At least three risk factors can be distinguished based on altered early Th1 lymphocyte development. First, the number of children with an inborn maturation defect may have increased since the beginning of the last century, when this condition would otherwise have had a lethal outcome without antibiotics and other modern health care (survival hypothesis). Second, another group of children in industrialized countries may have a deficit of environmental Th1 triggers during early life (hygiene hypothesis). A third factor may also be found predominantly in western societies. The prophylaxis of rickets with vitamin D has the apparent side effect of suppressing Th1 development (vitamin hypothesis).

Experimental as well as epidemiological studies now provide evidence for the vitamin hypothesis, which is examined in this paper by a time-course analysis of vitamin D application in Germany. Also paper studies in Swedish anthroposophic school children, the Tristan da Cunha islanders, and Swiss, Austrian and Bavarian farmers may be linked to either excessive or absent early vitamin D exposure.


*Clinical & Developmental Immunology*, June 2004, Vol. 11 (2), pp. 175–180

Taylor & Francis healthsciences

### The Triple T Allergy Hypothesis

MATTHIAS WJST*

*Gruppe Molekulare Epidemiologie, Institut für Epidemiologie, GSF - Forschungszentrum für Umwelt und Gesundheit, Ingolstädter Landstrasse 1, D-85758 Neuherberg, Munich, Germany*

The early induction of allergy is a complex process involving protective and destructive gene variants, environmental and nutritional co-factors as well as allergen exposure. Although critical doses, interactions and susceptible time frames have not been identified so far, late gestation and early childhood seem to be important time periods for allergic sensitization.

At least three risk factors can be distinguished based on altered early Th1 lymphocyte development. First, the number of children with an inborn maturation defect may have increased since the beginning of the last century, when this condition would otherwise have had a lethal outcome without antibiotics and other modern health care (survival hypothesis). Second, another group of children in industrialized countries may have a deficit of environmental Th1 triggers during early life (hygiene hypothesis). A third factor may also be found predominantly in western societies. The prophylaxis of rickets with vitamin D has the apparent side effect of suppressing Th1 development (vitamin hypothesis).

Experimental as well as epidemiological studies now provide evidence for the vitamin hypothesis, which is examined in this paper by a time-course analysis of vitamin D application in Germany. Also paper studies in Swedish anthroposophic school children, the Tristan da Cunha islanders, and Swiss, Austrian and Bavarian farmers may be linked to either excessive or absent early vitamin D exposure.

*Keywords*: Vitamin D; Th1 lymphocyte; T helper cells; Prophylaxis; Rickets; Allergy

Hundreds of studies have addressed the rise of allergies in industrialized countries. Research on risk factors has been inconclusive, factors associated in one study often failed confirmation in the next study (Kramer, 1988).

### THE TRIPLE T HYPOTHESIS

On a cellular level, it has been recognized that the increase in allergy might be a failure of the immune system to develop normal immune regulation, rather than a simple "Th2 skewing" of the immune response (Wills-Karp, *et al.*, 2001). Early T helper cell development seems to be severely disturbed in atopic children with low secretion of IFNγ (Holt *et al.*, 1992; Warner *et al.*, 1994; Prescott *et al.*, 1998). Th1 cells down-regulate Th2 cells in some systems. However, a simple Th1/Th2 paradigm has been questioned as it became clear that Th1 cells were themselves pro-inflammatory and could exacerbate allergic disease (Akbari *et al.*, 2003). Both conditions are not mutually exclusive and may coexist.

Based on the Th1/Th2 paradigm a dual T helper cell hypothesis of allergy initiation has been suggested (Wjst, 2004). The first line hypothesis comes from the population history of the last century. The survival of children with an inborn maturation defect—a genetic disposition—may have been increased by better health care since the beginning of the last century, when this condition would have been lethal (survival hypothesis), (Varner, 2002; Wjst, 2004). Second, another group of children may have a deficit of environmental Th1 triggers during early life, also termed the hygiene hypothesis (Prescott *et al.*, 2003). Third, prophylaxis of rickets with fortification of food in pregnant women and early vitamin D supplementation in newborns is an environmental suppressor of Th1 development (vitamin hypothesis) (Wjst and Dold, 1999). This paper will provide an update of the vitamin hypothesis and examine the time course of vitamin prophylaxis with the temporal relationship of the allergy epidemic.

### The Vitamin Hypothesis

Vitamin D was known to have endocrine effects in calcium homeostasis until two Science papers in the eighties showed the existence vitamin receptor also in peripheral mononuclear monocytes and provided

*Corresponding author.

ISSN 1740-2522 print/ISSN 1740-2530 online © 2004 Taylor & Francis Ltd
DOI: 10.1080/10446670410001722258

a novel immunoregulatory function (Provvedini *et al.*, 1983; Tsoukas *et al.*, 1984). The active metabolite of vitamin D, 1,25-dihydroxyvitamin D3, regulates the growth, differentiation and function of a broad range of cells, while the endocrine action is now seen as the "tip of the iceberg" (Feldman *et al.*, 1997) where most of the vitamin D effects are observed on dendritic cells. Differentiation of dendritic cells is suppressed with reduced surface marker expression, inhibited IL-12 and increased IL-10 production. Effects on other co-stimulatory molecules and apoptosis vary, (Cippitelli, 1998; Mathieu and Adorini, 2002; Adorini, 2003). One report showed vitamin D to be generally immunosuppressive (Pichler *et al.*, 2002), which is in accordance with the known operation of sunlight. Dendritic cell maturation appears to be impaired with reduced up-regulation of CD1a, HLA-DR, CD40, CD80, CD83 and CD86 (Piemonti *et al.*, 2000; Canning *et al.*, 2001; Penna and Adorini, 2001). Even a direct signaling of vitamin D on naïve CD4+ T cells toward Th2 differentiation or maintenance has been suggested (Cantorna *et al.*, 2000; Boonstra *et al.*, 2001; Mahon *et al.*, 2003).

Antigen-representing cell derived IL-12 and TNFα (in concert with Th1 cell-derived IFNγ) provide the main differentiation signals to T helper cells and stimulate the activity of T cytotoxic and NK cells. Interestingly, all these major cytokines—IL-12, TNFα and IFNγ—are regulated by vitamin D. IL-12 production is blocked by vitamin D (Lemire, 1995; D'Ambrosio *et al.*, 1998; Griffin *et al.*, 2001), and is antagonistic to the LPS action (Na *et al.*, 1999). This mechanism is not fully understood but it is known that the p40 gene promoter has an IFNγ-regulatory-factor 1 (IRF-1) binding site (Maruyama *et al.*, 2003). The reduced capacity of antigen-presenting cells to produce IL-12 in the perinatal period is in line with increased postnatal Th2 and diminished Th1 response (Prescott *et al.*, 2003). In contrast to IL-12, vitamin D increases TNFα mRNA in bone marrow macrophages in a dose- and time-dependent manner. It also binds to a vitamin D responsive element (VDRE) in the upstream promoter of TNFα (Hakim and Bar-Shavit, 2003). Consecutively, a lack of TNFα inhibits the development of allergic rhinitis in mice (Iwasaki *et al.*,2003), which is consistent with the observation that high human cord-blood concentrations of TNFα are associated with a lower risk of atopy (Macaubas *et al.*, 2003). Finally, vitamin D elicits an inhibitory action on IFNγ gene expression (Staeva-Vieira and Freedman, 2002). A repressive VDRE is also known in the IFNγ promoter (Cippitelli and Santoni, 1998). While a negative relationship between atopy and high IFNγ levels could be observed (Macaubas *et al.*, 2003). Taken together, there are three definite molecular pathways that link vitamin D exposure and allergy development.

Is the vitamin D effect persistent throughout lifetime? This may be concluded from experiments where immature dendritic cells maintain their status even after withdrawal of vitamin D and exhibit blunted responses to maturing stimuli like LPS (Griffin *et al.*, 2001). Another mechanism leading to a persistent state may be seen in the perpetuation of the initial expression of 25-hydroxyvitamin-D3-1-α-hydroxylase in monocyte-derived dendritic cells (Hewison *et al.*, 2003).

Another important question is whether vitamin D effects are also observed *in vivo* or only under experimental conditions. This could be answered in a recent animal study (Matheu *et al.*, 2003) where mice were immunized by intraperitoneal injection of chicken egg albumin and challenged on day 7 with the same allergen intranasally. By using another group pre-treated i.c. with 100 ng calcitriol they provoked higher allergen-specific IgE (600 ng/ml in the treatment group) than in the control group (450 ng/ml, $p < 0.05$) along with a cytokine profile typical for a Th2 reaction (Matheu *et al.*, 2003). Indirect evidence for *in vivo* effects also comes from human studies where insulin-dependent diabetes decreases with early vitamin D supplementation, an effect also described for multiple sclerosis, rheumatoid arthritis as well as Lyme arthritis, experimental allergic encephalomyelitis, thyroiditis, psoriasis and lupus nephritis (Hyppönen, 1999; Adorini, 2002; Mathieu and Adorini, 2002; Adorini, 2003). The effect on type 1 diabetes seems to last at least over the first 12 months of life (Stene and Joner, 2003).

So far there are only preliminary observations of vitamin D and later sensitization in humans. The first is more or less an anecdotal report that relates peanut oil used for the pharmacological preparation of vitamin D to peanut sensitization (de Montis *et al.*, 1993). The risk was remarkably high, with an odds ratio of (OR) of 9.0 ($p = 0.003$), for the vitamin supplement containing allergen compared to the supplement without allergen. A first human study has been performed in a Finnish birth cohort (Hyppönen, 2001). All births in the two northernmost provinces of Finland were recorded in 1966. Mothers reported frequency and dose of vitamin D supplementation when the infants were 1 year old ($n = 10,366$). Prevalence of atopy (OR 1.7, $p < 0.001$), hay fever (OR 1.5, $p < 0.001$) and asthma (OR 1.4, $p = 0.05$) at age 31 were all greater in participants with regular rather than irregular vitamin D supplementation (Hyppönen 2004; submitted). In an own observational birth cohort (LISA, München) the odds ratio for sensitization to any allergen tested at 24 months was 2.4 (0.6–10.0, Labereau, 2003; pers. comm.) where only 39 of 1754 children did not report vitamin D supplementation.

### Prophylaxis of Rickets and Rise of Allergy in Germany

Another important piece in the puzzle is the parallel increase of rickets and decrease of allergy. In 1923, rickets were still endemic: "Rickets occurs chiefly in Europe and North America. It is a disease found in cities. It is most prevalent in those nations whose wealth and industrial development have brought about most fully the, substitution of artificial conditions of living in place of the simple conditions which nature intended. Wherever civilization of this artificial character establishes new contacts, rickets begins to appear... The disease never occurs among people living under natural conditions. Savages may starve and may become the victims of pestilence, but they do not develop rickets. The disease does not occur in the native parts of Africa..." (Park, 1923). Rickets were consecutively recognized as a failure of newly formed osteoid to be mineralized, which is a vitamin D dependent process (Wharton and Bishop, 2003).

Although ergosterin had been discovered by Adolf Windaus in Göttingen (Germany) already in 1926 (awarded with the Nobel prize in chemistry in 1928), rickets was still a major problem in Germany for the following decades. In 1935, a dissertation (Riederer von Paar, 1936) examined 5632 live born children in Munich. The public welfare recorded that 38% of the children had signs of rickets. Mainly for costs reasons, prophylaxis was seldom used: a 10 ml Vigantol flask was sold for 1,35 Reichsmark and a complete prophylaxis cost 3,46. Reichsmark. In 1936, Harnapp at the Charité in Berlin introduced the "Stosstherapie", with up to 15 mg D2 (~600 IU) (Selle, 1987), which was recommended in 1939 by the "Reichsministerium des Innern" for prophylaxis ("Runderlass zur Rachitisprophylaxe"). A 10 mg single dose (Reinisch, 1989) developed as the prophylactic standard in high risk children born in autumn or already having a sibpair with rickets. The dose finding was "what has been the equivalent of a tablespoon liver cod oil", which had been proven safe for the prevention of rickets (Vieth, 1999).

During World War II vitamin prophylaxis was more or less discontinued and even in the postwar years vitamin D supply was difficult. In 1950 the state of Bavaria again introduced a single dose given by midwives ("Hebammenstoss"), which was then adopted also by other federal States. Around 1950, however, several reports of hypervitaminosis were published. This led Hermann Mai (1956), the Tübingen pediatrician, president of the German Society of Pediatrics and frequent visitor of Albert Schweitzers's Lambarene (where he never encountered rickets), to issue a warning on using the standard prophylactic scheme ("encourage caution") (Selle, 1987). His recommendation was 5 mg baseline vitamin given, fractionated into 5 single doses, with sufficient sunlight exposure and carefully control for the early signs of rickets. In contrast, Harnapp jun. continued with a 4 × 15 mg vitamin dose during the first 12 months, a scheme that was adopted in the centralized medical system of East Germany (since 7 Oct. 1949) and continued more or less until the fall of the wall (9 Nov. 1989). Doses of 15 mg (~100,000 IU) were given at months 1, 4, 7, 11, 15 and 20; in 1986 ergocalciferol was replaced by cholecalciferol.

The East German procedure was different from that in West Germany, where the situation from 1955–1965 was more or less chaotic. Von Giertmühlen in Hamburg introduced in 1960, a scheme of daily vitamin supplementation (von Giertmühlen, 1962), while Hellbrügge in Munich and Simon in Oldenburg recommended the fortification of milk (Reinisch, 1989). Some doctors treated pregnant women with two vitamin doses at months 6 and 9 (von Beuren, 1964), others used stoss therapy of the newborn, as single dose or fractionated, others prescribed daily vitamin doses from 300 to 5000 IU and others did nothing and waited for the first symptoms of rickets. Following reports of supravalvular aorta stenosis induced by hypervitaminosis, a press campaign started in West Germany accusing vitamin D as the culprit (von Beuren, 1964). Rickets increased again (Selle, 1987). As a consequence more and more physicians were in favor of daily low doses (Hövels, Reiss and others), a scheme that was finally adopted in West Germany following a symposium of the Society of Social Pediatrics 1970 in Karlsruhe, where a dose of 500 IU daily was recommended based on a large-scale study by Wolf and del Solar (1970) and Wolf *et al.* (1972). This procedure finally was adopted in West Germany; as of 1 July 1971 a central childhood examination program (Vorsorgeuntersuchung, U1–U6) was established. Rickets then did not play a major role in Germany: only 100 cases were observed over ten years in the isolated region of Berlin from 1978 to 1988, more than one-quarter were of Turkish nationality (26%) (Reinisch, 1991).

Unfortunately, there are no longitudinal data available on allergic sensitization that could be directly compared with the history of rickets prophylaxis. There is one cross-sectional study, however, that examined a large population census representative population where historical birth-cohorts may be constructed. Nicolai (1999) examined two large samples between 25 and 69 years of age in West (1991, $n = 5313$) and East Germany (1992, $n = 2617$) with a serological test screening for IgE antibodies to common aeroallergens. Results may not be over-interpreted as current sensitization may also depend on current risk factors (and birth cohorts are only available for longer time intervals). Nevertheless, a comparison may give an impression of whether there is any temporal relationship. At least 3 phases may be defined. In phase 1 cod liver oil was used (Sanostol and other preparations), with the first administration of synthetic vitamin D around 1940. Allergic sensitization is lowest in this cohort born around 1927, with ~5% sensitized both in East and West Germany. The 1937 and 1947 East and West cohorts showed a similar trend in sensitization prevalence of up to ~10%, which is in accordance with the more widespread use of vitamin D for prophylaxis. Phase 2 is characterized by a continuous "stoss" application in East Germany (DDR) from 1949 to 1989 (with a change of vitamin preparation in 1986) many ups and downs in West Germany (BRD) where after 1970 all children received daily vitamin doses. Correspondingly, the sensitization rate was stable in the East German cohort of 1947, all below 15%, while only the 1965 cohort showed a minor increase to ~18%. However, the most significant increase is seen in the West German cohort of 1957 (~23%), which even goes up to ~27% in the 1965 cohort. Phase 3 may be defined by the period after the unification of Germany where East German pediatricians abandoned their former vitamin D stoss therapy in favor of the West German daily application. The concomittant effect on allergic sensitization has been documented by many cross-sectional studies that showed initially much higher rates in West Germany, where the East German rate converges now on the VDU level (von Mutius, 1996). As a summary there seems to be a parallel trend in the German history of vitamin supplementation and increase of allergies.

### A CAUSAL RELATIONSHIP?

The geographical distribution of allergies has been reviewed earlier (Wjst and Dold, 1999). Where the vitamin D supplementation of milk and dairy products in many English-speaking countries is associated with high allergy rates (Salle, 2000), it is tempting to speculate that this dose is also sufficient to have immunological effects by its active metabolite. Mothers who received supplements during the third trimester had children that were on an average 400 g heavier and 1.6 cm longer at 12 months. Interestingly, the vitamin D receptor has been found in the fetus as early as the 13th week of gestation (Salle, 2000), while the allergen-specific response can be detected around the 22nd week (Jones, 1996).

Elegantly, the vitamin hypothesis also explains some other observations. It has been predicted that the increase of allergy in families with high socioeconomic status as well as the birth order effect may be explained by better adherence to vitamin prophylaxis recommendations (Wjst and Dodd, 1999). This could be shown in a Finnish birth cohort (Hyppönen 2003; pers. comm.). It has also been reported that being born abroad is better than being born in a high allergy country (Smith, 1976). Keeping the traditional lifestyle could have protected Turkish children born in Munich around 1980 from getting allergies like their native Bavarian peers (Kabesch, 1999), as Turkish immigrants did not come to Munich before 1961. Another interesting observation relates to the secular decrease of tuberculosis (Murray, 2004) where epidemiologic analysis also showed an inverse geographical relationship with allergy (von Mutius *et al.*, 2000). The vitamin hypothesis may provide the missing link as vitamin D deficiency is associated with susceptibility to tuberculosis (reviewed in Davies, 1985).

There are even more recent studies that may be interpreted in a different way than in their original context. One example is the Tristan da Cunha study with the unexpectedly high prevalence of allergy (Slutsky and Zamel, 1997). The islanders eat and process mainly sea bass (German Seebarsch or French Loup de Mer, N. Zamel, 2003; pers. comm.), known to be rich in vitamin D. The closely related striped bass contains approximately 1200 IU/ml liver oil (Sebrell, 1954). Another example is the East German margarine controversy, where margarine use has been associated with allergy in two studies (von Mutius, 1998; Bolte *et al.*, 2001). A 20 g margarine—the typical daily dose of one of the few foods supplemented in Germany with vitamin D—contains between 0 and 56 IU (S. Koch, 2003; pers. comm.) A nutritional supplement as used in the US may also explain the Boston “milk in crib” phenomenon (Celedon *et al.*, 2002) and also the recent Perth fish oil study of pregnant woman (Dunstan *et al.*, 2003). A lower allergy prevalence was observed in Swedish anthroposophic schools (Alm *et al.*, 1999), where parents avoid vitamin D. Finally, the protection of Swiss, Austrian and Bavarian farm children against allergies (Riedler, 1999) may be explained by neglected vitamin D supplementation due to consuming otherwise healthy home grown food.

Although some of the classical criteria of a causal relationship are already fulfilled, the final evidence could come from a large-scale prospective dose-titrating study of oral vitamin D supplementation that takes into account sunlight exposure as well as genetic vitamin sensitivity. It is an intriguing research question whether genetic polymorphisms in the metabolism and signaling pathway of vitamin D are modulating the vitamin response. In any case, vitamin D exposure is an elaborate factor in the triple T hypothesis of allergy induction.

### *References*

Adorini, L. (2003) “Tolerogenic dendritic cells induced by vitamin D receptor ligands enhance regulatory T cells inhibiting autoimmune diabetes”, *Ann. N Y Acad. Sci.* **987**, 258–261.

Adorini, L., Penna, G., Giarratana, N. and Uskokovic, M. (2003) “Tolerogenic dendritic cells induced by vitamin D receptor ligands enhance regulatory T cells inhibiting allograft rejection and autoimmune diseases”, *J. Cell. Biochem.* **88**, 227–233.

Akbari, O., Stock, P., DeKruyff, R.H. and Umetsu, D.T. (2003) “Role of regulatory T cells in allergy and asthma”, *Curr. Opin. Immunol.* **15**, 627–633.

Alm, J.S., Swartz, J., Lilja, G., Scheynius, A. and Pershagen, G. (1999) “Atopy in children of families with an anthroposophic lifestyle”, *Lancet* **353**(9163), 1485–1488.

Alroy, I., Towers, T.L. and Freedman, L.P. (1995) “Transcriptional repression of the interleukin-2 gene by vitamin D3: direct inhibition of the NFATp/AP-1 complex formation by a nuclear hormone receptor”, *Mol. Cell. Biol.* **15**, 5789–5799.

Bolte, G., Frye, C., Hoelscher, B., Meyer, I., Wjst, M. and Heinrich, J. (2001) “Margarine consumption and allergy in children”, *Am J Respir. Crit. Care Med.* **163**, 277–279.

Boonstra, A., Barrat, F.J., Crain, C., Heath, V.L., Savelkoul, H.F. and O’Garra, A. (2001) “1alpha,25-dihydroxyvitamin D3 has a direct effect on naive CD4(+) T cells to enhance the development of Th2 cells”, *J. Immunol.* **167**, 4974–4980.

Canning, M.O., Grotenhuis, K., de Wit, H., Ruwhof, C. and Drexhage, H.A. (2001) “1-alpha,25-Dihydroxyvitamin D3 (1,25(OH)(2)D(3)) hampers the maturation of fully active immature dendritic cells from monocytes”, *Eur. J. Endocrinol.* **145**, 351–357.

Cantorna, M.T., Humpal-Winter, J. and DeLuca, H.F. (2000) “*In vivo* upregulation of interleukin-4 is one mechanism underlying the immunoregulatory effects of 1,25-dihydroxyvitamin D(3)”, *Arch. Biochem. Biophys.* **377**, 135–138.

Celedon, J.C., Litonjua, A.A., Ryan, L., Weiss, S.T. and Gold, D.R. (2002) "Bottle feeding in the bed or crib before sleep time and wheezing in early childhood", *Pediatrics* **110**(6), e77.

Cippitelli, M. and Santoni, A. (1998) "Vitamin D3: a transcriptional modulator of the interferon-gamma gene", *Eur. J. Immunol.* **28**, 3017–3030.

Davies, P.D.O. (1985) "A possible link between vitamin D deficiency and impaired host defence to Mycobacterium tuberculosis", *Tubercle* **66**, 301–306.

D'Ambrosio, D., Cippitelli, M., Cocciolo, M.G., Mazzeo, D., Di Lucia, P., Lang, R., Sinigaglia, F. and Panina-Bordignon, P. (1998) "Inhibition of IL-12 production by 1,25-dihydroxyvitamin D3. Involvement of NF-kappaB downregulation in transcriptional repression of the p40 gene", *J. Clin. Investig.* **101**, 252–262.

de Montis, G., Gendrel, D., Chemillier-Truong, M. and Dupont, C. (1993) "Sensitisation to peanut and vitamin D oily preparations", *Lancet* **29**(8857), 341.

Dunstan, J.A., Mori, T.A., Barden, A., Beilin, L.J., Taylor, A.L., Holt, P.G. and Prescott, S.L. (2003) "Fish oil supplementation in pregnancy modifies neonatal allergen-specific immune responses and clinical outcomes in infants at high risk of atopy: a randomized, controlled trial", *J. Allergy Clin. Immunol.* **112**, 1178–1184.

Feldman, D., Wesley Pike, J. and Glorieux, F., eds (1997) Vitamin D (Academic Press).

Griffin, M.D., Lutz, W., Phan, V.A., Bachman, L.A., McKean, D.J. and Kumar, R. (2001) "Dendritic cell modulation by 1 alpha, 25 dihydroxyvitamin D3 and its analogs: a vitamin D receptor-dependent pathway that promotes a persistent state of immaturity *in vitro* and *in vivo*", *Proc. Natl Acad. Sci. USA* **98**, 6800–6805.

Hakim, I. and Bar-Shavit, Z. (2003) "Modulation of TNF-alpha expression in bone marrow macrophages: involvement of vitamin D response element", *J. Cell. Biochem.* **88**, 986–998.

Hewison, M., Freeman, L., Hughes, S.V., Evans, K.N., Bland, R., Eliopoulos, A.G., Kilby, M.D., Moss, P.A.H. and Chakraverty, R. (2003) "Differential regulation of vitamin D receptor and its ligand in human monocyte-derived dendritic cells", *J. Immunol.* **170**, 5382–5390.

Holt, P.G., Clough, J.B., Holt, B.J., Baron-Hay, M.J., Rose, A.H., *et al.* (1992) "Genetic risk for atopy is associated with delayed postnatal maturation of T-cell competence", *Clin. Exp. Allergy* **22**, 1093–1099.

Hyppönen, E., Laara, E., Reunanen, A., Jarvelin, M.R. and Virtanen, S.M. (2001) "Intake of vitamin D and risk of type 1 diabetes: a birth-cohort study", *Lancet* **358**, 1500–1503.

Iwasaki, M., Saito, K., Takemura, M., Sekikawa, K., Fujii, H., Yamada, Y., Wada, H., Mizuta, K., Seishima, M. and Ito, Y. (2003) "TNF-alpha contributes to the development of allergic rhinitis in mice", *J. Allergy Clin. Immunol.* **112**, 134–140.

Jones, A., Miles, E., Warner, J., Colwell, B., Bryant, T. and Warner, J. (1996) "Fetal peripheral blood mononuclear cell proliferate responses to mitogenic and allergenic stimuli during gestation", *Pediatr. Allergy Immunol.* **7**, 109–116.

Kabesch, M., Schaal, W., Nicolai, T. and von Mutius, E. (1999) "Lower prevalence of asthma and atopy in Turkish children living in Germany", *Eur. Respir. J.* **13**, 577–582.

Kramer, M.S. (1988) "Does breast feeding help protect against atopic disease? Biology, methodology, and a golden jubilee of controversy", *J. Pediatr.* **112**, 181–190.

Lemire, J.M. (1995) "Immunomodulatory actions of 1,25-dihydroxy-vitamin D3", *J. Steroid. Biochem. Mol. Biol.* **53**, 599–602.

Macaubas, C., de Klerk, N.H., Holt, B.J., Wee, C., Kendall, G., Firth, M., Sly, P.D. and Holt, P.G. (2003) "Association between antenatal cytokine production and the development of atopy and asthma at age 6 years", *Lancet* **362**(9391), 1192–1197.

Mahon, B.D., Wittke, A., Weaver, V. and Cantorna, M.T. (2003) "The targets of vitamin D depend on the differentiation and activation status of CD4 positive T cells", *J. Cell. Biochem.* **89**, 922–932.

Maruyama, S., Sumita, K., Shen, H., Kanoh, M., Xu, X., Sato, M., Matsumoto, M., Shinomiya, H. and Asano, Y. (2003) "Identification of IFN regulatory factor-1 binding site in IL-12 p40 gene promoter", *J. Immunol.* **170**, 997–1001.

Matheu, V., Bäck, O., Mondoc, E. and Issazadeh-Navikas, S. (2003) "Dual effects of vitamin D-induced alteration of TH1/TH2 cytokine expression: enhancing IgE production and decreasing airway eosinophilia in murine allergic airway disease", *J. Allergy Clin. Immunol.* **112**, 585–592.

Mathieu, C. and Adorini, L. (2002) "The coming of age of 1,25-dihydroxyvitamin D(3) analogs as immunomodulatory agents", *Trends Mol. Med.* **8**, 174–179.

Murray, J.F. (2004) "Mycobacterium tuberculosis and the cause of consumption. From discovery to fact", *Am. J. Resp. Crit. Care. Med.* **169**, 1086–1088.

Na, S.Y., Kang, B.Y., Chung, S.W., Han, S.J., Ma, X., Trinchieri, G., Im, S.Y., Lee, J.W. and Kim, T.S. (1999) "Retinoids inhibit interleukin-12 production in macrophages through physical associations of retinoid X receptor and NFkappaB", *J. Biol. Chem.* **274**, 7674–7680.

Nicolai, T., Bellach, B., Mutius, E.V., Thefeld, W. and Hoffmeister, H. (1997) "Increased prevalence of sensitization against aeroallergens in adults in West compared with East Germany", *Clin. Exp. Allergy* **27**, 886–892.

Park, E.A. (1923) "The etiology of rickets", *Physiol. Rev.* **3**, 106–163.

Penna, G. and Adorini, L. (2001) "Inhibition of costimulatory pathways for T-cell activation by 1,25-dihydroxyvitamin D(3)", *Transplant. Proc.* **33**, 2083–2084.

Pichler, J., Gerstmayr, M., Szepfalusi, Z., Urbanek, R., Peterlik, M. and Willheim, M. (2002) "1 alpha,25(OH)2D3 inhibits not only Th1 but also Th2 differentiation in human cord blood T cells", *Pediatr. Res.* **52**, 12–18.

Piemonti, L., Monti, P., Sironi, M., Fraticelli, P., Leone, B.E., Dal Cin, E., Allavena, P. and Di Carlo, V. (2000) "Vitamin D3 affects differentiation, maturation, and function of human monocyte-derived dendritic cells", *J. Immunol.* **164**, 4443–4451.

Prescott, S.L., Macaubas, C., Holt, B.J., Smallacombe, T.B., Loh, R., Sly, P.D. and Holt, P.G. (1998) "Transplacental priming of the human immune system to environmental allergens: universal skewing of initial T cell responses toward the Th2 cytokine profile", *J. Immunol.* **160**, 4730–4737.

Prescott, S.L., King, B., Strong, T.L. and Holt, P.G. (2003) "The value of perinatal immune responses in predicting allergic disease at 6 years of age", *Allergy* **58**, 1187–1194.

Provvedini, D.M., Tsoukas, C.D., Deftos, L.J. and Manolagas, S.C. (1983) "1,25-dihydroxyvitamin D3 receptors in human leukocytes", *Science* **221**, 1181–1183.

Reinisch, M. "Eine epidemiologische Studie über das Auftreten von Rachitis im Zeitraum 1978–1988 in der Stadt Berlin (West) unter besonderer Berücksichtigung neuerer Literatur zum Vitamin D und zur Problematik der Rachitisprophylaxe und der Gefahr der Intoxikation", Dissertation (Berlin).

Riederer von Paar, V. "Über die Rachitis in München im Jahre, 1935", Dissertation (München).

Riedler, J., Braun-Fahrländer, C., Eder, W., Schreuer, M., Waser, M., Maisch, S., Carr, D., Schierl, R., Nowak, D. and von Mutius, E. (2001) "Exposure to farming in early life and development of asthma and allergy: a cross-sectional survey", *Lancet* **358**(9288), 1129–1133.

Salle, B.L., Delvin, F.E., Lapillonne, A., Bishop, N.J. and Glorieux, F.H. (2000) "Perinatal Metabolism of vitamin D", *Am. J. Clin. Nutr.* **71**(suppl. 5), 13175–13245.

Sebrell, W.H. (1954) The Vitamins: Chemistry, Physiology, Pathology, Methods (Academic Press).

Selle, B., "Rachitis und Spasmophilie: Ein Überblick über 75 Jahre an Hand der Krankenblätter des Kaiserin Auguste Victoria Hauses 1987", Dissertation (Berlin).

Slutsky, A.S. and Zamel, N. (1997) "Genetics of asthma: the University of Toronto Program University of To ronto Genetics of Asthma Research Group", *Am. J. Respir. Crit. Care Med.* **156**, S130–S132.

Smith, J.M. (1976) "The prevalence of asthma and wheezing in children", *BMJ* **70**, 73–77.

Staeva-Vieira, T.P. and Freedman, L.P. (2002) "1,25-dihydroxyvitamin D3 inhibits IFN-gamma and IL-4 levels during in vitro polarization of primary murine CD4+ T cells", *J. Immunol.* **168**, 1181–1189.

Strachan, D.P. (1989) "Hay fever, hygiene, and household size", *Br. Med. J.* **299**, 1259–1260.

Tsoukas, C.D., Provvedini, D.M. and Manolagas, S.C. (1984) "1,25-dihydroxyvitamin D3: a novel immunoregulatory hormone", *Science* **224**, 1438–1440.

Varner, A.E. (2002) "The increase in allergic respiratory diseases. Survival of the fittest?", *Chest* **121**, 1308–1316.

Vieth, R. (1999) "Vitamin D supplementation, 25-hydroxyvitamin D concentrations, and safety", *Am. J. Clin. Nutr.* **69**, 842–856.

von Beuren, A.J., Schulze, C.H., Eberle, P., Harmjanz, D. and Apitz, J. (1964) "The syndrome of supravalvular aortic stenosis, peripheral pulmonary stenosis,mental retardation and similar facial appearance", *Am. J. Cardiol.* **13**, 471–483.

von Giertmühlen, F. (1962) "Rachitisprophylaxe und Therapie in den letzten vier Jahrzehnten", *Münchner Med. Wschr.* **45**, 2192–2196.

von Mutius, E., Weiland, S.K., Fritzsch, C., Duhme, H. and Keil, U. (1998) "Increasing prevalence of hay fever and atopy among children in Leipzig, East Germany", *Lancet.* **351**(9106), 862–866.

von Mutius, E., Pearce, N., Beasley, R., Cheung, S., von Ehreustein, O., Björksten, B. and Weiland, S. (2000) "International patterns of tuberculosis and the prevalence of symptoms of asthma, rhinitis, and eczema", *Thorax* **55**, 449–453.

Warner, J.A., Miles, E.A., Jones, A.C., Quint, D.J., Colwell, B.M. and Warner, J.O. (1994) "Is deficiency of interferon gamma production by allergen triggered cord blood cells a predictor of atopic eczema?", *Clin. Exp. Allergy* **24**, 423–430.

Wharton, B. and Bishop, N. (2003) "Rickets", *Lancet* **362**(9393), 1389–1400.

Wills-Karp, M., Santelitz, J. and Karp, C.L. (2001) "The germless theory of allergic disease: revisiting the hygiene hypothesis", *Nat. Rev. Immunol.* **1**, 69–75.

Wjst, M. (2004) "Is the increase in allergic asthma associated with an inborn Th1 maturation or with an environmental Th1 trigger defect?", *Allergy* **169**, 1217–1223.

Wjst, M. and Dold, S. (1999) "Genes, factor X and allergens: what causes allergic diseases?", *Allergy* **54**(7), 757–759.

Wolf, H. and del Solar, E. (1970) "Rachitisprophylaxe beim Säugling", *Dtsch med Wschr* **29**, 1530.

Wolf, H., Kerstan, J. and Kreutz, F.H. (1972) "Kontinuierliche Rachitisprophylaxe — schon beim Neugeborenen?", *Monatsschrift für Kinderheilkunde* **120**, 329–333.

